# Flood risk assessment for residences at the neighborhood scale by owner/occupant type and first-floor height

**DOI:** 10.3389/fdata.2022.997447

**Published:** 2023-01-09

**Authors:** Ayat Al Assi, Rubayet Bin Mostafiz, Carol J. Friedland, Md Adilur Rahim, Robert V. Rohli

**Affiliations:** ^1^Bert S. Turner Department of Construction Management, Louisiana State University, Baton Rouge, LA, United States; ^2^LaHouse Resource Center, Department of Biological and Agricultural Engineering, Louisiana State University Agricultural Center, Baton Rouge, LA, United States; ^3^Coastal Studies Institute, Louisiana State University, Baton Rouge, LA, United States; ^4^Department of Oceanography and Coastal Sciences, College of the Coast and Environment, Louisiana State University, Baton Rouge, LA, United States; ^5^Engineering Science Program, Louisiana State University, Baton Rouge, LA, United States

**Keywords:** flood risk, average annual loss (AAL), natural hazard mitigation, Louisiana, Hazus Multi-Hazard (Hazus-MH), first-floor height (FFH), owner/occupant type, neighborhood resilience

## Abstract

Evaluating flood risk is an essential component of understanding and increasing community resilience. A robust approach for quantifying flood risk in terms of average annual loss (AAL) in dollars across multiple homes is needed to provide valuable information for stakeholder decision-making. This research develops a computational framework to evaluate AAL at the neighborhood level by owner/occupant type (i.e., homeowner, landlord, and tenant) for increasing first-floor height (FFH). The AAL values were calculated here by numerically integrating loss-exceedance probability distributions to represent economic annual flood risk to the building, contents, and use. A simple case study for a census block in Jefferson Parish, Louisiana, revealed that homeowners bear a mean AAL of $4,390 at the 100-year flood elevation (*E*_100_), compared with $2,960, and $1,590 for landlords and tenants, respectively, because the homeowner incurs losses to building, contents, and use, rather than only two of the three, as for the landlord and tenant. The results of this case study showed that increasing FFH reduces AAL proportionately for each owner/occupant type, and that two feet of additional elevation above *E*_100_ may provide the most economically advantageous benefit. The modeled results suggested that Hazus Multi-Hazard (Hazus-MH) output underestimates the AAL by 11% for building and 15% for contents. Application of this technique while partitioning the owner/occupant types will improve planning for improved resilience and assessment of impacts attributable to the costly flood hazard.

## 1. Introduction

Floods are among the most severe and frequently occurring natural disasters (Tariq and van de Giesen, 2012; Sastry, 2021; Mostafiz et al., 2022a) and cause significant human and economic losses (Tate et al., 2014). For example, direct damage from flooding in the U.S.A. has increased to $17 billion per year (Association of State Floodplain Managers, 2020). More than 1.1 million insurance claims were filed with the National Flood Insurance Program (NFIP) between 1998 and 2017 (Matthews et al., 2021). Further, the total average annual loss (AAL) from floods, presumably due to direct and indirect causes that are either insured or uninsured, is estimated at $104 billion worldwide (Eder et al., 2022) and $32.1 billion in the U.S.A. (Wing et al., 2022). In the next 30 years, flood-related property damage may increase by 60% as a result of climate change (Sastry, 2021), and many communities and individuals underestimate the potential for flood damage (Burningham et al., 2008; Mol et al., 2020). Therefore, many researchers worldwide have focused on flood risk and loss assessments (e.g., Dutta et al., 2003; Scawthorn et al., 2006; Tam et al., 2014; Afifi et al., 2019; Mostafiz et al., 2021a,d; Al Assi et al., 2022a,b; Jin et al., 2022; Rahim et al., 2022a,b) and the value of mitigating the flood risk (Taghinezhad et al., 2020).

Raising the first-floor height (FFH) above the 100-year flood elevation (*E*_100_) is one of the most successful flood mitigation strategies (Taghinezhad et al., 2020). In the U.S.A., improvements in mitigating the flood hazard have involved the establishment of minimum construction elevations and increasingly active encouragement to build above the minimum height. The base flood elevation (BFE), which is approximately equal to *E*_100_ or the 1% annual exceedance probability (AEP; Federal Emergency Management Agency, 2011), is the national standard used by the NFIP and all federal agencies (Federal Emergency Management Agency, 2005). The regulatory standard for home construction in areas where wave heights are <1.5 feet (known in the U.S.A. as the V Zone and Coastal A Zone) has typically been to situate the top of the first floor at the *E*_100_ (ASCE, 2005), a standard that was modified (ASCE, 2014) to include an additional 1.0 foot of elevation above *E*_100_. Elevating the home above *E*_100_ leads to reduced building and contents damage by decreasing the probability of flood occurrence above the first floor (Hawkesbury-Nepean Floodplain Management Steering Committee, 2007). However, little information is available that describes the benefits of elevation for different owner/occupant type.

Flood risk assessments consist of two main components: the probability of flooding and the consequences associated with its occurrence (Dalezios, 2017). Recent studies quantify AAL to represent the flood risk (Dunn, 2004; Arnbjerg-Nielsen and Fleischer, 2009; Federal Emergency Management Agency, 2013; Montgomery and Kunreuther, 2018; Armal et al., 2020; Zarekarizi et al., 2020). In its representation of flood risk, AAL includes the costs associated with three types of losses considered in previous flood loss research (e.g., Scawthorn et al., 2006; National Institute of Building Sciences, 2017; Taghinezhad et al., 2020): (1) restoring the building structure itself to its pre-flood fair market value (i.e., direct building loss); (2) replacing flood-damaged physical contents inside the building with items of the same fair market value (i.e., direct contents loss); and (3) accounting for the time and labor required for the inhabitant to repair, clean up, and inspect the building, beyond those associated with (1) and (2) above (i.e., indirect loss), represented here by loss of use. These losses vary between homeowners, landlords, and tenants (Hamideh et al., 2018; Warren-Myers et al., 2018; Friedland et al., 2022). However, most residential flood risk assessment research to date focuses solely on building and contents risk to homeowners; risk to the landlord and tenant owner/occupant types have largely been overlooked, leading to inaccurate flood risk estimates.

Although such approaches have been accepted to evaluate flood AALs, recent research (Gnan et al., 2022a) has identified substantial limitations and showed that application of a refined numerical integration approach addresses these limitations. Specifically, this refined numerical integration approach models the risk across the full range of exceedance probabilities, in contrast with other approaches that rely only on available data, such as Federal Emergency Management Agency (2013) and Armal et al. (2020). However, Gnan et al. (2022a) has shown that risk estimates vary widely based on the damage initiation point of the depth damage function (DDF) that describes the relationship between the water depth in the structure (*dh*) and loss.

Flood risk assessment has been performed at many scales, from the micro- (Gnan et al., 2022b,c) to the meso- (Lüdtke et al., 2019) and macro- (Olsen et al., 2015; Armal et al., 2020). Although studies at these scales enable people and communities to prepare most effectively for floods and develop risk maps, some limitations need to be addressed. For example, the total macro-scale residential flood damage calculated by Armal et al. (2020) as AAL and by Olsen et al. (2015) as expected annual damage lacks specifics regarding damage to buildings, contents, and loss of use. Likewise, at the meso-scale, residential losses calculated by Lüdtke et al. (2019) are presented as aggregated data without considering the building inventory and categorization of damage. At the micro-scale, Gnan et al. (2022a) apply the refined numerical integration approach to predict AAL considering home elevation value in flood risk reduction, but this approach has only been attempted for a one-story, single-family home considering only direct building and content losses. None of the studies described above take into account the owner/occupant type (i.e., homeowner vs. landlord vs. tenant). A need remains for research that provides a comprehensive framework for estimating the flood risk for residential buildings at the neighborhood level and presents the results on a per-building and aggregated basis, while considering the various owner/occupant types and the value of home elevation.

This paper develops a framework that evaluates the flood risk across multiple homes with various combinations of building attributes, first floor elevation, and owner/occupant type (i.e., homeowner, landlord, and tenant), expanded in scope to also consider loss of use risk to demonstrate how the following research questions can be answered: (1) How does flood risk vary by owner/occupant type (i.e., homeowners vs. landlords vs. tenants)? (2) What is the mean flood risk reduction by owner/occupant type with increasing FFH above an initial first-floor height (*FFH*_0_)? and (3) What effect does the DDF damage initiation point at a flood depth of zero vs. at negative flood depths, which could damage the building foundation and/or basement, have on AAL and therefore flood risk estimates? To demonstrate how these questions can be addressed, the AAL, partitioned by loss type (i.e., building, contents, and use), is calculated by owner/occupant type (i.e., homeowner, landlord, and tenant). For each combination of loss type and owner/occupant type, the reduction of AAL is calculated by increasing FFH above *FFH*_0_ in one-foot increments up to four feet. A sensitivity analysis is conducted to assess the effect of choosing the damage initiation point on each AAL estimation. Calculations from this approach are compared to those generated by the Hazus Multi-Hazard (Hazus-MH) Flood Model (Federal Emergency Management Agency, 2013).

The contribution of this research is the development of a computational framework that implements the refined numerical integration approach to enable assessment of flood risk across multiple homes by loss type, owner/occupant type, and FFH. A simple case study demonstrates this framework, while allowing evaluation of individual building results. Results are displayed on a per-building and aggregated basis to describe the neighborhood-level risk, with individual results available for further scrutiny. In addition to evaluate the flood risk across multiple homes, understanding the absolute and relative economic effectiveness of FFH by owner/occupant type supports hazard planning and mitigation to decrease flood risk. Application of the improved, refined numerical integration approach across multiple homes with varying attributes is useful in future work for assessing community-level flood risk (Mostafiz et al., 2022b), thereby leading to more informed decision-making (Mostafiz et al., 2022c) at the second-most-local scale in the spectrum.

## 2. Methodology

**Figure 1:**
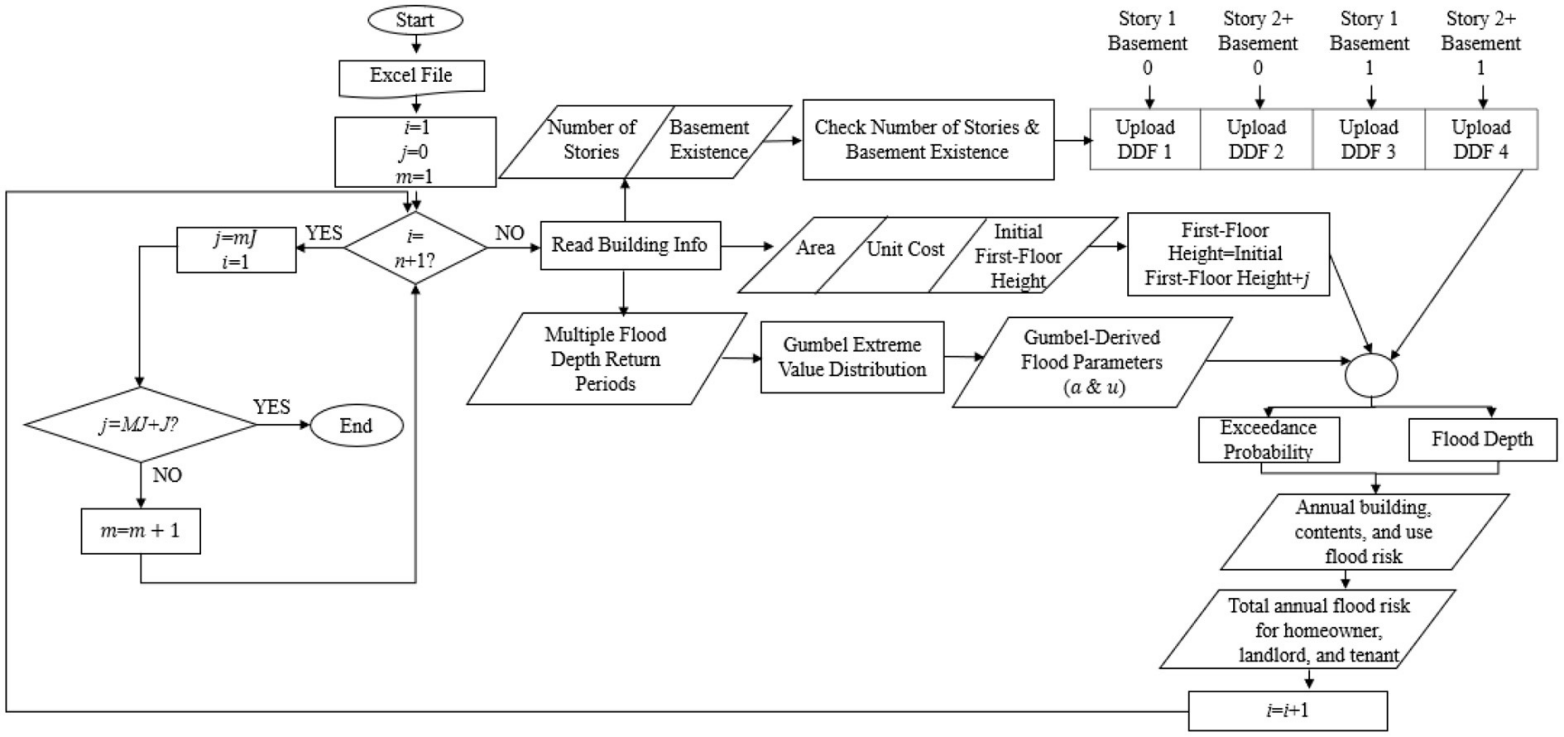
General outline of the research framework, for homes (i to n), targeted elevation (j) at each step, constant increment by which the elevation increases (J) for each step, number of steps (M), and final targeted elevation (MJ).

To address the research questions, a novel computational framework is developed (Figure 1) using the MATLAB R2019b software package to estimate AAL for multiple homes distributed across a spatial extent with varying flood hazard. The AAL is partitioned to homes separately for building, contents, and use. Likewise, the AAL reduction (in dollars) is calculated at each increment of FFH above the *FFH*_0_, for each owner/occupant type (i.e., homeowner, landlord, and tenant). These results are compared with AAL calculations using Level 2 analysis in the Hazus-MH Flood Model (Federal Emergency Management Agency, 2013).

### 2.1. Input data

The input data (e.g., Supplementary Table 1) used in the AAL analysis are the number of stories (1 or 2+), basement existence (0 = No, 1= Yes), area in square feet (*A*), unit area repair cost in USD per square foot (*C*_*R*_), and *FFH*_0_. *FFH*_0_ is a general term that may represent either the actual FFH for existing buildings, the minimum regulatory standard for FFH for a particular area, a hypothetical beginning FFH for consideration of additional elevation, or a practical minimum FFH based on foundation type. Also, the flood depth above the ground for multiple return periods at each home and the corresponding AEP values for those flood depths are input.

The DDF selection is a critical decision for flood loss, and therefore risk assessment (Mostafiz et al., 2021b). The most effective DDF will characterize the relationship between the water depth in the structure (*dh*) and the percent of damage most appropriately while also identifying properly the flood damage initiation point with respect to *dh*. Several sources examine the *dh*-damage relationship, including United States Army Corps of Engineers (2000), United States Army Corps of Engineers (2006), and Nofal et al. (2020) who provided loss functions for multiple models, and Wing et al. (2020) who used NFIP flood damage claims to represent the *dh*-damage relationships.

In this study, the United States Army Corps of Engineers (2000) DDFs are chosen to determine the relationship between building (or contents) loss *L*_*B*_ (and separately, *L*_*C*_) and *dh*. These DDFs are selected in this study to demonstrate the methodology because they are based on generic data (1996–2000). DDFs such as United States Army Corps of Engineers (2006) include flood duration and foundation type and could be substituted in cases for which more descriptive input data are available. Four of the United States Army Corps of Engineers (2000) home types are incorporated into the numerically integrated computational framework here: one story with no basement (DDF1), two or more stories with no basement (DDF2), one story with basement (DDF3), and two or more stories with basement (DDF4). Within the DDF, the mean and standard deviation of building and contents loss are expressed as a proportion of home replacement cost value (*V*_*R*_) for each one-foot increment of *dh* beginning at the damage initiation point of −2 feet, up to 16 feet (United States Army Corps of Engineers, 2000). Interpolation is used here to determine the percentage of building and contents losses at each 0.5-foot increment of *dh*. Finally, restoration time (in months) is taken from Federal Emergency Management Agency (2013) as a surrogate for use loss (*L*_*U*_), at each flood depth. Because restoration time tables in Federal Emergency Management Agency (2013) define *L*_*U*_ at four-foot increments, interpolation is employed to estimate *L*_*U*_ at 0.5-foot increments of *dh*. The four utilized DDFs are shown in Supplementary Tables 2–5. The *DDF* selected for each home corresponds to the number of stories and presence/absence of a basement.

### 2.2. Flood risk quantification

The Gumbel distribution function has been shown to be effective for modeling flood frequency (e.g., Singh et al., 2018; Patel, 2020; Rahim et al., 2021). Therefore, the two-parameter Gumbel distribution function is used to model the probability of exceedance for the expected flood depths. The cumulative distribution function (CDF) of the Gumbel distribution is the probability (*p*) that a random variable *X* has a value less than or equal to a threshold *d*, also known as the probability of non-exceedance. That value is generally expressed in Equation 1 (Patel, 2020), with *u* and *a* representing the Gumbel location and scale parameter, respectively.


(1) $$\begin{array}{l} CDF=p\left(X\leq d\right)=\exp\left[-exp\left(-\left(\frac{d-u}{a}\right)\right)\right] \end{array}$$

The complement of the CDF represents the *AEP* of a potential flood event with depth above the ground *d*, as shown in Equation 2.


(2) $$\begin{array}{l} AEP=P=1-\text{exp}\left[-exp\left(-\left(\frac{d-u}{a}\right)\right)\right] \end{array}$$

where *P* = *p*(*X*>*d*)

Solving Equation 1 for *d* yields Equation 3, which represents the relationship between *d* and the double natural logarithm of *p*, where *a* and *u* are the regression coefficients (slope and y-intercept, respectively). Using the flood depth (*d*) data and corresponding double natural logarithm of *p* for each return period, the Gumbel distribution is fit and a unique regression line is generated for each home location to yield its distinctive *a* and *u* hazard parameters (Mostafiz et al., 2021c, 2022d). The flood depth within the house, represented in the DDFs as *dh*, is expressed as the difference between *d* and *FFH*_0_ (Equation 4).


(3) $$\begin{array}{lll} d & = & \;u\;-\;a\;\ln\left(-ln\left(p\right)\right) \end{array}$$

(4) $$\begin{array}{lll} dh & = & \;d\;-\;FFH_{0} \end{array}$$

For each 0.5-foot water depth increment of *dh*, the corresponding *d* is calculated using Equation 4. The generated flood parameters and the calculated *d* are used to calculate the corresponding *P* using Equation 3. For each home, the selected DDFs are transformed into a function of *P* using the relationships in Equations 2, 3. The functions *L*_*B*_(*P*) and *L*_*C*_(*P*) represent building and contents losses as a function of *P*, expressed as a proportion of the *V*_*R*_. Similarly, *L*_*U*_(*P*) represents use loss in months as a function of *P*. AAL is calculated as the integral of loss as a function of flood probability, where *P*_min_ represents the lowest exceedance probability value and *P*_max_ is the highest exceedance probability value. Equations 5–7 describe the theoretical formulation of the AAL for building, contents, and use (*AAL*_*B*/_*V*__*R*__, *AAL*_*C*/_*V*__*R*__, and *AAL*_*U, months*_, respectively) for each home. *AAL*_*B*/_*V*__*R*__ and *AAL*_*C*/_*V*__*R*__ represent the annual building and contents flood risk as a proportion of the *V*_*R*_, while *AAL*_*U, months*_ represents annual use flood risk in months.


(5) $$AAL_{B/V_{R}}=\int_{P_{\min}}^{P_{\max}}L_{B}\left(P\right)dP\;$$

(6) $$AAL_{C/V_{R}}=\int_{P_{\min}}^{P_{\max}}L_{C}\left(P\right)dP\;$$

(7) $$AAL_{U,months}=\int_{P_{\min}}^{P_{\max}}L_{U}\left(P\right)dP\;$$

Riemann summation is a computational approach to approximate an exact integration solution as the sum of trapezoidal areas under a curve. To evaluate Equations 5–7 computationally, the area of each trapezoid under the *L*(*P*) functions is estimated as the difference in exceedance probabilities multiplied by the average loss (building, contents, or use) for the corresponding probabilities. The trapezoidal Riemann sums approach is used to aggregate the product results across all probabilities to yield AAL (Meyer et al., 2009; Gnan et al., 2022a) as generally shown in Equation 8.


(8) $$\begin{array}{l} AAL=\overset{K}{\underset{k=1}{\sum }}\left[\left(P_{k+1}-P_{k}\right)*\frac{\left(L_{k}+L_{k+1}\right)\;}{2}\right] \end{array}$$

Here, *K* is the number of trapezoids under the *L*(*P*) curve.

### 2.3. Flood risk quantification by owner/occupant type

The total AAL calculations vary based on the owner/occupant type (i.e., homeowner, landlord, or tenant). Any building risk will always be incurred by the owner (i.e., homeowner or landlord), regardless of occupancy. Homeowners and tenants, but not landlords, will incur contents risk. For this study, AALs for building and contents are initially expressed as a proportion of the *V*_*R*_ (Equations 5, 6). However, because monetary values are understood and appreciated more readily when communicating risk to the public, the AAL variables are then converted to dollar figures for building (*AAL*_*B$*_) and contents (*AAL*_*C$*_) *via* *V*_*R*_, which is the unit building replacement cost per square foot (*C*_*R*_) multiplied by the home area (*A*), as shown in Equation 9:


(9) $$\begin{array}{l} V_{R}=C_{R}\times A \end{array}$$

Then, *AAL*_*B$*_ and *AAL*_*C$*_ are calculated as shown in Equations 10, 11.


(10) $$\begin{array}{l} AAL_{B\$}=\;AAL_{B/V_{R}}\times V_{R} \end{array}$$

(11) $$\begin{array}{l} AAL_{C\$}=\;AAL_{C/V_{R}}\times V_{R} \end{array}$$

The AAL for use, or restoration time, is expressed in units of months (*AAL*_*U, months*_) and is partitioned into that assumed by the homeowner (*AAL*_*UH, months*_), landlord (*AAL*_*UL, months*_), and tenant (*AAL*_*UT, months*_). These three variables are then converted to economic value, as shown in Equations 12–14, where *R*_*l*_ is the monthly rent incurred (for the homeowner) or lost (for the landlord), and *H*_*R*_ is the nightly hotel rent. The total annual flood risk (*AAL*_*T$*_) calculated as the sum of building, contents, and use annual risk by owner/occupant type (Equation 15). *AAL*_*U$*_ is a generalized representation of use risk for which the applicable equation should be selected based on owner/occupant type. Table 1 shows the equation numbers to use when calculating *AAL*_*B$*_, *AAL*_*C$*_, and *AAL*_*U$*_ for each owner/occupant type.


(12) $$\begin{array}{lll} AAL_{UH,\$} & = & \;AAL_{UH,months}\times \;R_{l} \end{array}$$

(13) $$\begin{array}{lll} AAL_{UL,\$} & = & \;AAL_{UL,months}\times R_{l} \end{array}$$

(14) $$\begin{array}{lll} AAL_{UT,\$} & = & \;AAL_{UT,months}\times {30\;}_{days/month}\times H_{R} \end{array}$$

(15) $$\begin{array}{lll} AAL_{T\$} & = & \;AAL_{B\$}+\;AAL_{C\$}+AAL_{U\$} \end{array}$$

**Table 1:** Equation numbers used to calculate building, contents, and use average annual loss (*AAL*_*B$*_, *AAL*_*C$*_, and *AAL*_*U$*_) by owner/occupant type.

**Owner/occupant type**	***AAL*_**B*$*_**	***AAL*_**C*$*_**	***AAL*_**U*$*_**
Homeowner	10	11	12
Landlord	10	n/a	13
Tenant	n/a	11	14

### 2.4. Relative flood risk reduction with increasing first-floor height

To calculate the AAL for FFH above *FFH*_0_, an increment (*j*) is added to *FFH*_0_ and all calculations represented in Equations 3–15 are repeated using the new FFH value. The relative AAL reduction with each increment is calculated using Equation 16.


(16) $$AAL\;Reduction_{j}\;\%=\frac{\left(AAL_{FFH_{0}})\;-\;(AAL_{FFH_{0}+j}\;\right)}{AAL_{FFH_{0}}}*100$$

### 2.5. Average annual loss (AAL) in Hazus-MH

Flood depth grids at 10-, 25-, 50-, 100-, and 500-year return periods are required to calculate “average annualized loss.” Hazus-MH represents AAL *via* summed calculations of the product of difference in return period (*RP*) flood frequency (*f*_*RP*_; analogous to AEP) and the corresponding mean loss (*L*_*RP*_), as shown in Equation 17.


(17) $$\begin{array}{l} AAL=\left(f_{10}-f_{25}\right)\;\cdot \frac{L_{10}+L_{25}\;}{2}+\left(f_{25}-f_{50}\right)\;\cdot \frac{L_{25}+L_{50}\;}{2}\\ \;+\left(f_{50}-f_{100}\right)\;\cdot \frac{L_{50}+L_{100}\;}{2}+\left(f_{100}-f_{500}\right)\;\cdot \frac{L_{100}+L_{500}\;}{2}\\ \;+\left(f_{500}\cdot \;L_{500}\right) \end{array}$$

The process begins when “create a new region” is selected, with “riverine only” clicked in the study region flood hazard type. Then, the 10-, 25-, 50-, 100-, and 500-year flood depth grids under “user data” are uploaded. Next, the building input data are imported under “user defined facilities (UDF).” The proper DDFs are then selected for the flood loss calculation. Then, a new hazard scenario is created with the 10-, 25-, 50-, 100-, and 500-year flood depth grids, and the riverine floodplain is delineated for “full suite of return periods” using the default cell size (3.048 × 3.048 m). Then, AAL analysis is run with the UDF. The result shows *AAL*_*B$*_ and *AAL*_*C$*_ for each building separately, but Hazus-MH does not provide *AAL*_*U$*_.

### 2.6. DDF sensitivity

**Figure 2:**
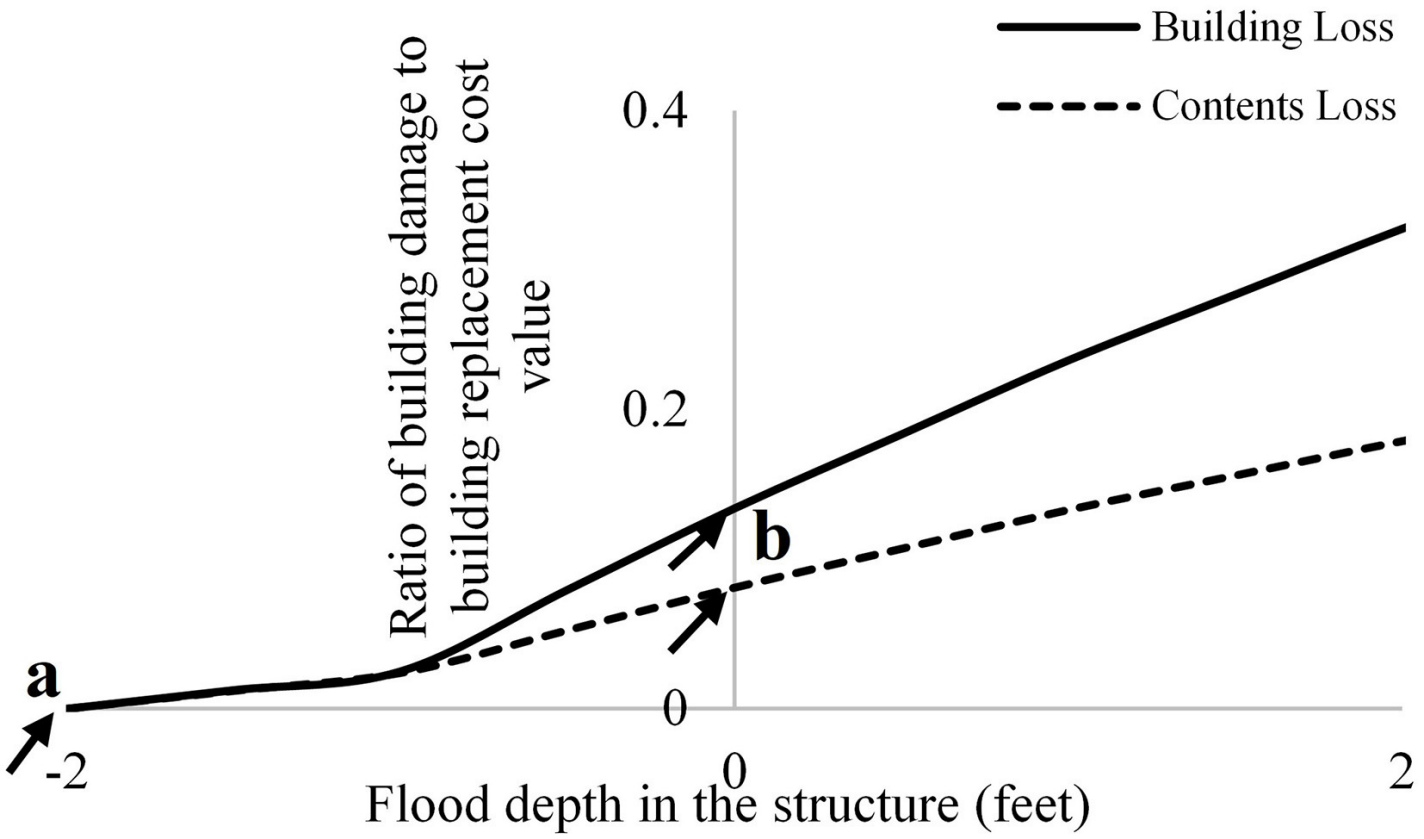
Depiction of United States Army Corps of Engineers (2000) mean depth-damage function for a one-story, no-basement residential building, starting at flood depth of **(a)** −2 feet (original function), and **(b)** 0 feet.

The *dh* is an important parameter for all flood loss models (Apel et al., 2009), in part because it is used in the DDFs to estimate building loss (*L*_*B*_) and contents losses (*L*_*C*_); and therefore total AAL (Pistrika et al., 2014). More recent research has highlighted the importance of *dh* where damage is assumed to initiate. Gnan et al. (2022a) demonstrated that AAL derived from USACE functions that initiate damage at a *dh* of −2 feet (i.e., 2 feet below the top of the first floor) were much higher than AAL calculated from the same function with damage initiation assumed to occur at *dh* = 0 (i.e., top of first floor; Figure 2). Consideration of flood risk reduction through optimal home elevation and the sensitivity of risk assessment to DDF selection remain as fundamental issues in the development of consistent flood risk assessment procedures.

Flood waters can damage subfloor assemblies and utilities of elevated homes, which may require consideration of losses for *dh* < 0. However, the extent to which these losses may occur is unclear. A sensitivity analysis is therefore conducted to compare AAL values estimated using United States Army Corps of Engineers (2000) considering damage initiation points of *dh* = −1 foot, −0.5 foot, and 0 feet, holding all input parameters and the DDF constant. While this sensitivity analysis does not identify which damage initiation point is “correct,” it does shine light on the effect of DDF damage initiation point variability on flood risk assessment results.

### 2.7. Comparison with Hazus-MH results

A pairwise *t*-test is used to assess whether a statistically significant difference (α = 0.05) exists in population mean (μ) values between *AAL*_*B$*_ (and in a separate analysis, *AAL*_*C$*_) calculated by the AAL approach presented in this paper vs. Hazus-MH output. Because Hazus-MH ignores use risk, *AAL*_*U$*_ is not considered in this analysis. Thus, the null and alternative hypotheses for both *AAL*_*B$*_ and *AAL*_*C$*_ are provided in Equations 18, 19, respectively. The intent of this test is to evaluate whether the refined approach presented in this paper differs significantly from the results of the existing, widely used Hazus-MH tool.


(18) $$\begin{array}{lll} H_{0}:\;\mu_{AAL} & = & \;\mu_{AAL\;Hazus} \end{array}$$

(19) $$\begin{array}{lll} {H_{1}:\mu }_{AAL} & \neq & \mu_{AAL\;Hazus} \end{array}$$

## 3. Case study

Several criteria should be met by the case study selected in order to address the research questions most effectively. The selected area should be subjected to flooding and be densely populated, so that flood impacts are detectable. Availability of high-quality flood hazard data at multiple return periods and building data for each home in the study area is also essential. The study area should also be sufficiently large and diverse to allow for meaningful calculations and transferability of results elsewhere, yet small enough to ensure uniformity in flood hazard, which promotes improved generalizability of FFH results across space.

**Figure 3:**
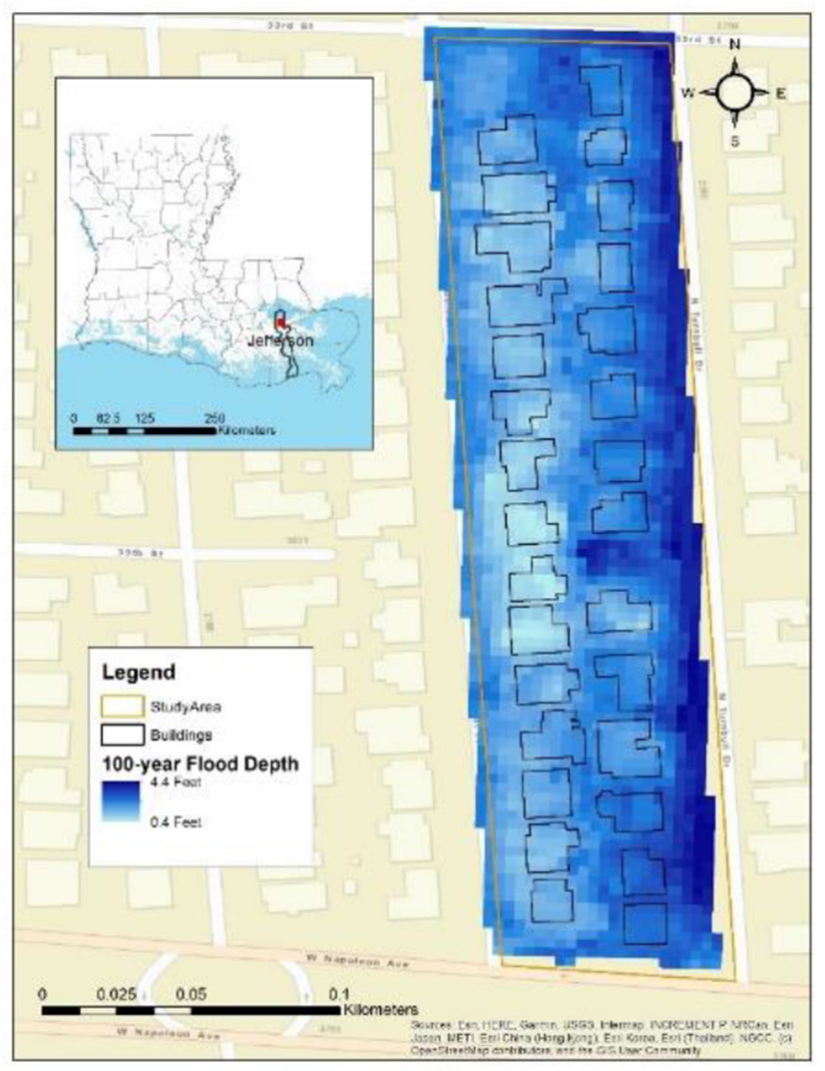
Location of the study area with non-numbered building footprint polygons.

Based on these criteria, census block 220510220012004, in Metairie, Louisiana, U.S.A., which consists of *n* = 29 homes, none of which have basements, is selected (Figure 3). The building shapefiles and 0.10, 0.02, 0.01, and 0.002 AEP flood depth grids are collected from the governmental office (Jefferson Parish, 2022). To protect the privacy of homeowners, landlords, and tenants, numbering of homes within the study area is randomized and not disclosed. The input data—area, unit cost, *FFH*_0_ (assumed to be equal to *E*_100_), number of stories, basement existence, and 0.10, 0.02, 0.01, and 0.002 AEP flood depth point values—are used to calculate the *AAL*_*B$*_, *AAL*_*C$*_, *AAL*_*UH, $*_, *AAL*_*UL, $*_, *AAL*_*UT, $*_, and *AAL*_*T*_ for each home. Input parameters for each home, along with values for the four AEP flood depths, are listed in Supplementary Table 1; values for other AEPs can be provided upon request. To evaluate the effects of FFH, increases in FFH from 1 to 4 feet (i.e., *J* through *MJ*) are added to *E*_100_. The same input parameters are analyzed using Hazus-MH with United States Army Corps of Engineers (2000) DDFs and a flood depth damage initiation point at −1 foot, for comparison with results from the presented AAL approach.

## 4. Results

### 4.1. Flood risk quantification

**Figure 4:**
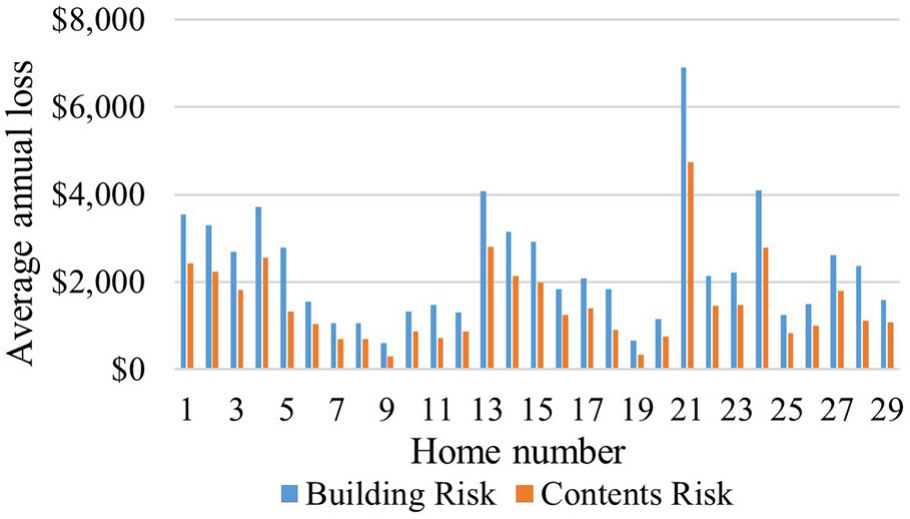
Building and contents AAL at 100-year flood elevation, by home.

Flood risk mean values for the 29 homes in this case study represent neighborhood-level values. For homes with FFH equal to the 100-year flood elevation, the mean (i.e., neighborhood-level) *AAL*_*B$*_ and *AAL*_*C$*_ is $2,300 and $1,500 per home, respectively, with a range of ~$600–$7,000 and $300–$4,700 per home, respectively (Figure 4). Table 2 shows that the building attributes, *V*_*R*_, and regression parameters differ substantially for the homes with the largest (i.e., $11,636) and smallest (i.e., $894) AAL values.

**Table 2:** Building attributes, initial first-floor height, replacement cost value, and regression parameters for residences with the largest and smallest total AALs for building + contents.

**Building number**	**Number of stories**	**Basement**	***FFH*_0_ (feet) = *E*_100_**	**Replacement cost value ($)**	***a***	***u***	**Total building + contents loss**
21	1	0	0.6	283,804	0.29	−0.64	$11,636
9	2	0	2.7	195,129	0.56	−0.03	$894

**Figure 5:**
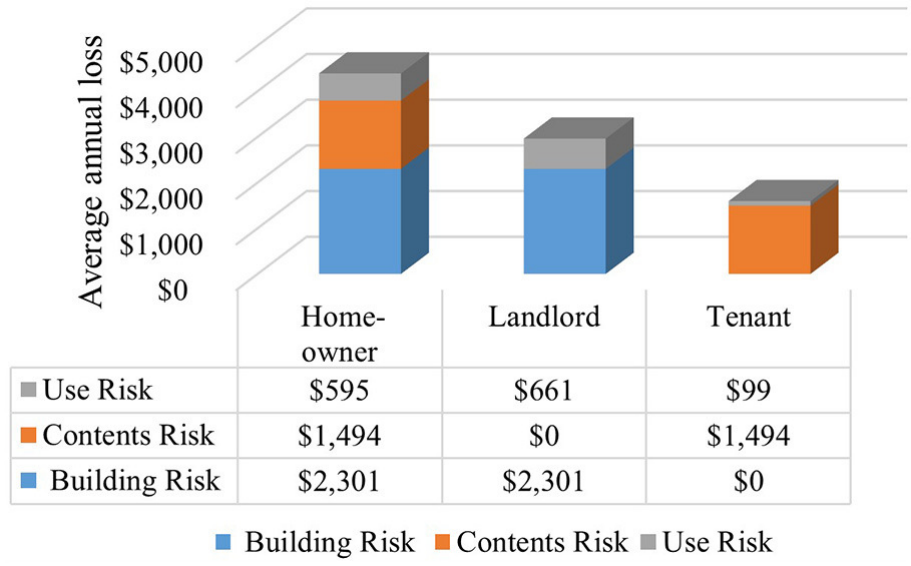
AAL for building, contents, and use by owner/occupant type at 100-year flood elevation, if all homes would be in the same owner/occupant type.

Because the *AAL*_*U$*_ calculation depends on owner/occupant type (i.e., homeowner, landlord, and tenant), it is not shown in aggregate form. As owner/occupant type is unknown, the sum of *AAL*_*B$*_, *AAL*_*C$*_, and *AAL*_*U$*_ for homes at *E*_100_ assumes that all 29 homes are homeowner-occupied (and then landlord-owned and tenant-occupied), yielding a mean *AAL*_*T$*_ of $4,390, $2,960, and $1,590 per home, respectively (Figure 5). The complete set of output values for each home in the study area is shown in Supplementary Table 6.

### 4.2. Flood risk reduction quantification

**Figure 6:**
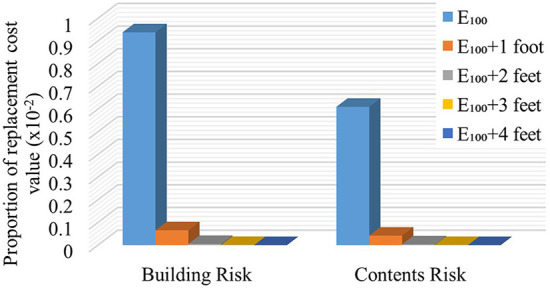
Mean *AAL*_*B*/_*V*__*R*__ and *AAL*_*C*/_*V*__*R*__ by increasing FFH above 100-year flood elevation.

The mean *AAL*_*B*/_*V*__*R*__ (and separately, *AAL*_*C*/_*V*__*R*__) for the 29 homes for each increase in FFH above *E*_100_ is shown in Figure 6. The calculated *AAL*_*B$*_, *AAL*_*C$*_, and *AAL*_*U$*_ by owner/occupant type and for each increase in FFH above *E*_100_ appear in Table 3. Substantial decreases in all components of AAL are realized with a 1-foot increase in FFH above *E*_100_, and more modest decreases in AAL occur with additional increases above *E*_100_, for all owner/occupant types (Table 4).

**Table 3:** Mean *AAL*_*B$*_, *AAL*_*C$*_, and *AAL*_*U$*_ (USD) per home by increasing FFH above 100-year flood elevation.

**FFH**	**Building AAL**	**Contents AAL**	**Use AAL homeowner**	**Use AAL landlord**	**Use AAL tenant**
	**($)**	**($)**	**($)**	**($)**	**($)**
*E*_100_ + 0 feet	2,300	1,494	595	661	99
*E*_100_ + 1 foot	157	101	42	47	7
*E*_100_ + 2 feet	12	8	3	4	<1
*E*_100_ + 3 feet	1	<1	<1	<1	<1
*E*_100_ + 4 feet	<1	<1	<1	<1	<1

**Table 4:** *AAL*_*T*_ reduction by combination of increases in FFH above 100-year flood elevation and owner/occupant type.

**FFH**	***AAL*_*T, Homeowner*_**	***AAL*_*T, Landlord*_**	***AAL*_*T, Tenant*_**
*E*_100_ + 1 foot	93.156%	93.111%	93.267%
*E*_100_ + 2 feet	99.475%	99.450%	99.482%
*E*_100_ + 3 feet	99.949%	99.947%	99.952%
*E*_100_ + 4 feet	99.999%	99.999%	99.999%

### 4.3. DDF sensitivity

The means of *AAL*_*B$*_ and sum of *AAL*_*B$*_ and *AAL*_*C$*_ increase ~6-fold, and *AAL*_*C$*_ increases 7-fold, when the damage initiation point is considered at *dh* = −1.0 foot vs. 0 feet (Table 5). Similarly, use of DDFs starting at *dh* = −1.0 foot causes a 5-fold increase in mean *AAL*_*T*_ (i.e., building, contents, and use) for homeowner and landlord, and a 6-fold increase for tenant, compared with *AAL*_*T*_ at *dh* = 0 feet (Table 6).

**Table 5:** Sensitivity of mean of the average annual loss values to building and contents (USD) per home to depth-damage function damage initiation point.

***dh* (feet)**	***AAL*_*B$*_ ($)**	***AAL*_*C$*_ ($)**	**Sum ($)**
0	374	217	591
−0.5	1,004	626	1,630
−1	2,300	1,493	3,793

**Table 6:** Sensitivity of the average annual loss mean values (USD) per home for homeowner, landlord, and tenant to depth-damage function damage initiation point.

***dh* (feet)**	***AAL*_*T, Homeowner, $*_ ($)**	***AAL*_**T, Landlord*, $*_ ($)**	***AAL*_*T, Tenant, $*_ ($)**
0	836	646	258
−0.5	2,270	1,710	725
−1	4,390	2,960	1,590

### 4.4. Comparison with Hazus-MH

A test of the null hypotheses equating mean AAL with Hazus-MH output results in *p*-values of 0.39 and 0.23 for building and contents, respectively. These results indicate that no statistically significant difference exists between the mean *AAL* value from the presented approach vs. results from Hazus for both building and contents risk. The mean *AAL*_*B$*_ and *AAL*_*C$*_ are $2,330 and $1,513 per home, respectively, for the numerically integrated approach. In contrast, these values are $2,069 and $1,279 per home based on Hazus output.

## 5. Discussion

### 5.1. Flood risk quantification

The wide variation in AAL across the case study area, with homes 21 and 9 having the highest and lowest *AAL*_*B$*_ and *AAL*_*C$*_ (Figure 4), demonstrates the value of performing neighborhood-level risk assessment. Many factors affect AAL results. Further analysis to interpret the results presented in Supplementary Table 6 demonstrates that unique DDFs based on number of stories result in statistically significant differences in *AAL*_*C$*_ to *AAL*_*B$*_ ratios (*p*-value of 0.0004), with mean values of 67 and 49% for one- and two-story homes, respectively. Other factors, such as *a* and *u* parameters, *FFH*_0_, and *V*_*R*_ also affect AAL (Table 2). The finding that *AAL*_*T$*_ for homeowner-occupied homes exceeds that for the other owner/occupant types (Figure 5) is reasonable because the homeowner bears the risk for building, contents, and use, while the smallest *AAL*_*T$*_ is borne by tenants because they incur no *AAL*_*B$*_.

### 5.2. Flood risk reduction quantification

Quantification of the flood risk is important to overcome the resistance to mitigate the flood hazard (Hollar, 2017). Results from assessing flood risk by owner/occupant type and the effect of increasing FFH on its reduction can enhance awareness and action to mitigate flood effects. For example, the substantial decrease in *AAL*_*B$*_ and *AAL*_*C$*_ by increasing the FFH 1 foot above *E*_100_ (Figure 6 and Table 3) and the virtual elimination of building and contents flood risk through elevation of 4 feet, shown in this case study, demonstrate the economic advantages of mitigation measures and flood risk reduction. The reduction of AAL by ~93% with the first foot of increase in FFH above *E*_100_, with an additional 6, 0.48, and 0.05% with two through four feet of additional elevation (Table 4), regardless of owner/occupant type, provides the public with specific information for decision-making. These values suggest that adding two feet above *E*_100_ may provide the most effective flood risk reduction for this study area. However, it is noted that the substantial flood risk prevented by adding one foot above the *E*_100_ is achieved by the minimum elevation requirement for residential buildings in the U.S.A. specified in the ASCE (2014) technical standard.

These results vary by DDF damage initiation point and completely depend on the accuracy of flood maps, particularly regarding the flood depth information for multiple return periods. Error can also be incurred if the modeling of these data excludes recent changes in development that affect rainfall runoff conditions or climatic data not reflective of the true nature of precipitation events. Future work is needed to understand the extent to which the finding of 93% reduction with first foot of increase in FFH above *E*_100_ and an additional 6% with two feet elevation is consistent across space and time. Regardless, however, presentation of flood risk in terms of AAL and the corresponding reduction in relative flood risk with additional building information provides new opportunities to validate flood model data and DDFs through observation of future events and losses.

### 5.3. DDF sensitivity

Consideration of the damage initiation point at *dh* = −1 and −0.5 foot increases the quantified risk substantially, as suggested by Tables 5, 6. Given the 5- to 7-fold increase in AAL calculation based solely on the DDF damage initiation point, this concept warrants considerable future research, as values of *dh* = −2 (United States Army Corps of Engineers, 2000, 2003), *dh* = −1 (FIA, 1974), and *dh* = 0 (Federal Emergency Management Agency, 2003) are employed in the literature. DDFs are available that differentiate foundation type and floodwater characteristics and duration [e.g., (Gulf Engineers and Consultants (GEC), 1997)], and standard deviation values are provided for generalized United States Army Corps of Engineers (2000) DDFs. As micro-scale analysis gains more traction, the use of component-based functions (Matthews et al., 2021) may become viable to generate building-specific DDFs. However, while the current research represents a substantial step forward, without more research in this area it is likely that flood risk estimates continue to underestimate or overestimate the true risk.

### 5.4. Comparison with Hazus-MH

**Figure 7:**
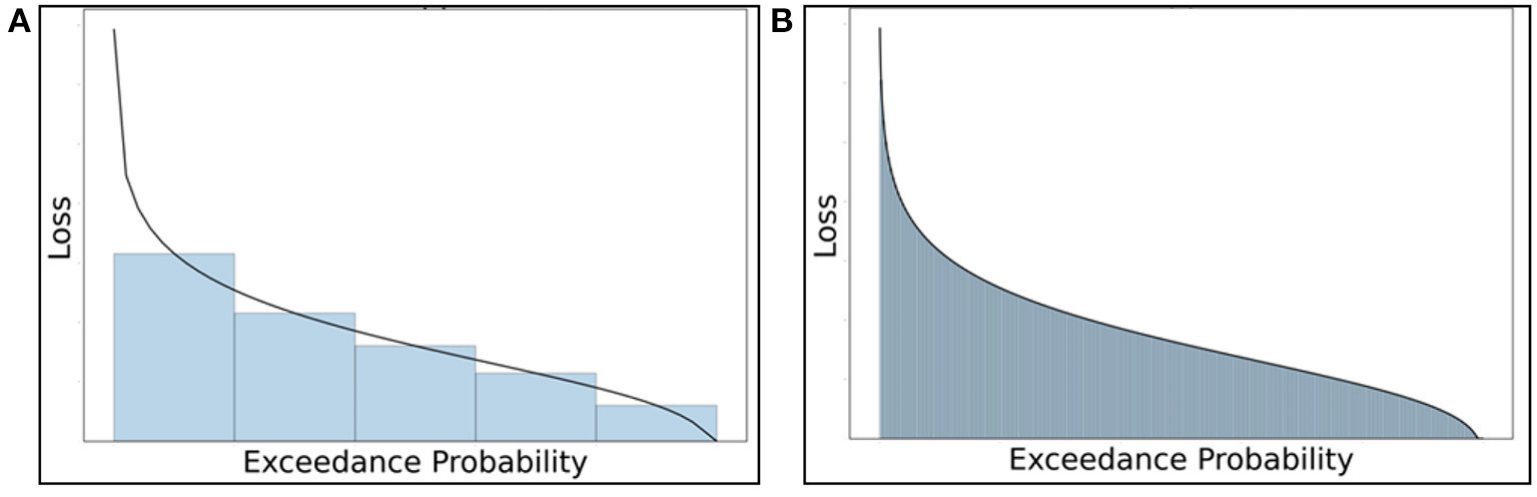
Comparison of coarseness using the Riemann sum approach: **(A)** coarse trapezoidal areas under the loss-exceedance probability curve in the Hazus-MH approach vs. **(B)** fine trapezoids used in the proposed AAL approach.

Despite the fact that the Hazus-MH does not differ significantly from the refined numerical integration approach-generated mean AALs, an 11 and 15% on average underestimation of *AAL*_*B$*_ and *AAL*_*C$*_, respectively, by Hazus in the case study results may be crucial for an individual homeowner. The underestimation can be attributed to two factors. First, Hazus-MH does not consider flooding at shorter than 10-year and longer than 500-year return periods (Equation 17), which leaves damage from the most common, minor floods, and the rarest, major floods, unconsidered. While at first glance such floods may be negligible either because they produce little damage or because they are unlikely to occur during the home's useful life cycle, the frequency with which nuisance floods occur and the devastating impacts of exceedingly large floods, if they do happen to occur during the home's useful life cycle, make consideration of flood risk only for return periods ranging from 10 to 500 years incomplete. A second factor contributing to the Hazus-MH underestimate is that it only considers five return periods, which generates a coarser approximation using Riemann sums of areas under the loss-exceedance curve (Figure 7A) vs. the fine trapezoid definition used in this AAL approach, where 260 trapezoids are used (Figure 7B).

In addition to the building and contents underestimates, other factors point to further inaccuracies by Hazus-MH. The neglect of use risk can generate inaccuracies, especially for longer-term recoveries. Moreover, the final term in Equation 17 (from Hazus-MH), unrepresented in Figure 7A, is an additional factor without theoretical basis that may ostensibly compensate for the underestimation, perhaps leading to the finding that the AAL approach differs insignificantly from Hazus-MH.

## 6. Conclusion

Accurate AAL estimation and sound policies and planning based on that estimation enable mitigation of flood risk to a known and tolerable level. Also, quantifying flood risk at the neighborhood level aids in selecting the most cost-effective and beneficial technique for mitigating future flood hazards. This study develops an approach to neighborhood-level flood risk estimation that assists in identifying the home elevation that reduces flood risk most efficiently for three owner/occupant types.

Home attribute data, DDFs, and *FFH*_0_ are combined with flood depth above the ground for multiple annual exceedance probability (AEP) events each home location. Using the Gumbel extreme value distribution, flood hazard regression parameters (i.e., *a* and *u*) enable quantification of flood depth and probability of exceedance. These, in turn, are used to calculate the AAL expressed as a proportion of home *V*_*R*_ for building, contents, and use. The approach is applied to a case study of 29 residences in Metairie, Louisiana, with different combinations of attributes and owner/occupant types. Sensitivity analyses are conducted to examine the effect of DDF damage initiation point on flood risk assessment results. Also, the reduction of AAL with increasing FFH above *E*_100_, by owner/occupant type, is computed. Then, AAL for building and contents are compared with those from Hazus-MH. General conclusions are:

- Using the refined numerically integrated approach enhances AAL estimates by addressing several limitations of other approaches.
- Home attributes such as number of stories, basement existence, *FFH*_0_, area, unit cost, and multiple return period flood depths affect AAL calculations.
- Analyzing a large number of homes may provide a clearer understanding of community flood risk.

Specific conclusions, based on the case study, are:

- The ratio between contents and building AAL for single-family residences without a basement is higher for one-story than two-or-more-story homes.
- Increasing the FFH by one foot above *E*_100_ results in ~90% flood risk reduction. However, increasing the FFH by two feet above *E*_100_ may provide the most economically advantageous benefit, at nearly 99% flood risk reduction.
- Homeowner AAL ($4,390) exceeds that of landlord ($2,960), with tenant AAL ($1,590) being lowest. Although flood insurance implications are not considered, this paper serves as an important methodological step that may facilitate more robust consideration of insurance scenarios by owner/occupant type.
- The DDF damage initiation point has a large impact on the AAL calculations. AAL is increased 5- to 7-fold if the DDF damage initiation point is considered at *dh* = −1 foot vs. *dh* = 0. Therefore, future research defining the proper damage initiation point is essential.
- Although this AAL approach produces statistically insignificant mean differences from those generated by Hazus-MH for *AAL*_*B$*_ and *AAL*_*C$*_, an 11% and 15% on average underestimation of *AAL*_*B$*_ and *AAL*_*C$*_, respectively, by Hazus may be important for an individual homeowner. Moreover, Hazus incorporates an additional, theoretically unfounded term that may compensate for several sources of underestimation, and Hazus fails to incorporate *AAL*_*U$*_; it appears that the AAL approach improves representation of flood risk.

This study suggests that application of the proposed refined numerical integration approach that considers the full range of loss-exceedance probabilities enhances the accuracy of AAL estimation. In the present research, loss of use is considered, which represents a substantial step forward from previous analyses, but loss of use is only one component of indirect loss. Future research should focus on further consideration of indirect and intangible losses, which are important flood loss metrics to consider when understanding the impacts of floods on residents. Landlord contents loss should be considered more explicitly, as well as the assumptions made about homeowner costs for temporary and longer-term lodging after a flood. This paper serves as the basis for future integration of flood insurance considerations to reduce flood risk by owner/occupant type.

## Data availability statement

The original contributions presented in the study are included in the article/Supplementary material, further inquiries can be directed to the corresponding author.

## Author contributions

AA developed the methodology, analyzed the data, interpreted the findings, developed the initial text, and revised the manuscript in response to reviewer comments. RM selected the case study area, prepared the input data, contributed to the analysis, and supervised the research. CF conceptualized the research idea, supervised the research, provided insight and recommendation for the research, and reviewed and edited the manuscript. MR verified the results and reviewed the manuscript. RR provided insight and recommendation for the research, reviewed and edited the manuscript, and assisted in revising the manuscript in response to reviewer comments. All authors contributed to the article and approved the submitted version.
