# Supplementary material for: Flood risk assessment for residences at the neighborhood scale by owner/occupant type and first-floor height
Source: Front Big Data. 2023 Jan 9;5:997447. doi: 10.3389/fdata.2022.997447 (PMC9868865; doi:10.3389/fdata.2022.997447)
Supplement: Supplementary file 1 [file Table_1.docx]

**Supplementary Tables**

**Supplementary Table 1.** Input Parameters for the 29 Homes in the Metairie, Louisiana, Study Area.

| **Building Number** | **Area**  **(sq. ft.)** | **Unit cost per square foot ($)** | **Number of stories** | **Basement** | ${FFH}_{0}$ **(feet)** | | **0.10 AEP** | **0.02 AEP** | **0.01 AEP** | **0.002 AEP** |
| --- | --- | --- | --- | --- | --- | --- | --- | --- | --- | --- |
| 1 | 2791 | 110.3438 | 1 | 0 | 1.0 | 0.2 | | 0.6 | 1.0 | 1.4 |
| 2 | 2436 | 110.3438 | 1 | 0 | 1.4 | 0.6 | | 1.3 | 1.4 | 2.1 |
| 3 | 2028 | 110.3438 | 1 | 0 | 0.9 | 0.1 | | 0.7 | 0.9 | 1.5 |
| 4 | 2938 | 110.3438 | 1 | 0 | 1.4 | 0.6 | | 1.0 | 1.4 | 1.8 |
| 5 | 2181 | 106.222 | 2 | 0 | 1.2 | 0.5 | | 1.1 | 1.2 | 1.9 |
| 6 | 2420 | 110.3438 | 1 | 0 | 2.0 | 1.1 | | 1.7 | 2.0 | 2.4 |
| 7 | 1677 | 110.3438 | 1 | 0 | 2.1 | 1.2 | | 1.8 | 2.1 | 2.6 |
| 8 | 1749 | 110.3438 | 1 | 0 | 2.1 | 1.2 | | 1.8 | 2.1 | 2.5 |
| 9 | 1837 | 106.222 | 2 | 0 | 2.7 | 1.8 | | 2.3 | 2.7 | 3.1 |
| 10 | 2308 | 110.3438 | 1 | 0 | 2.4 | 1.6 | | 2.1 | 2.4 | 2.9 |
| 11 | 2582 | 106.222 | 2 | 0 | 1.8 | 1.0 | | 1.4 | 1.8 | 2.3 |
| 12 | 1695 | 110.3438 | 1 | 0 | 1.8 | 0.7 | | 1.5 | 1.8 | 2.3 |
| 13 | 1951 | 110.3438 | 1 | 0 | 0.7 | 0.1 | | 0.5 | 0.7 | 1.3 |
| 14 | 1914 | 110.3438 | 1 | 0 | 0.8 | 0.1 | | 0.6 | 0.8 | 1.4 |
| 15 | 2258 | 110.3438 | 1 | 0 | 1.3 | 0.5 | | 1.0 | 1.3 | 1.8 |
| 16 | 2306 | 110.3438 | 1 | 0 | 1.6 | 0.8 | | 1.1 | 1.6 | 1.9 |
| 17 | 2327 | 110.3438 | 1 | 0 | 1.7 | 1.1 | | 1.3 | 1.7 | 2.1 |
| 18 | 3458 | 106.222 | 2 | 0 | 2.1 | 1.4 | | 1.8 | 2.1 | 2.7 |
| 19 | 1795 | 106.222 | 2 | 0 | 2.6 | 1.8 | | 2.2 | 2.6 | 3.0 |
| 20 | 1757 | 110.3438 | 1 | 0 | 2.2 | 1.5 | | 1.9 | 2.2 | 2.7 |
| 21 | 2572 | 110.3438 | 1 | 0 | 0.6 | 0.1 | | 0.5 | 0.6 | 1.3 |
| 22 | 1645 | 110.3438 | 1 | 0 | 1.3 | 0.5 | | 1.0 | 1.3 | 1.8 |
| 23 | 2613 | 110.3438 | 1 | 0 | 1.7 | 0.9 | | 1.4 | 1.7 | 2.2 |
| 24 | 3090 | 110.3438 | 1 | 0 | 1.1 | 0.3 | | 0.9 | 1.1 | 1.7 |
| 25 | 1590 | 110.3438 | 1 | 0 | 1.8 | 1.0 | | 1.5 | 1.8 | 2.3 |
| 26 | 2073 | 110.3438 | 1 | 0 | 2.1 | 1.5 | | 1.8 | 2.1 | 2.6 |
| 27 | 2062 | 110.3438 | 1 | 0 | 1.0 | 0.2 | | 0.6 | 1.0 | 1.4 |
| 28 | 2320 | 106.222 | 2 | 0 | 1.4 | 0.6 | | 1.2 | 1.4 | 2.0 |
| 29 | 1490 | 110.3438 | 1 | 0 | 1.5 | 0.6 | | 1.2 | 1.5 | 2.0 |

**Supplementary Table 2.** Relationship between Flood Depth, Proportion of Building (or Content) Value Damaged (Represented as Building Loss and Contents Loss, Respectively), and Restoration Time (Represented as Use Loss) for Each Owner/Occupant Type, for a One-Story Home without Basement ($\mathrm{DDF}$1).

| **From USACE (2000)** | | | **From FEMA (2013)** | | |
| --- | --- | --- | --- | --- | --- |
| **Flood Depth in Structure**  **(**$\boldsymbol{dh}$**, in feet)** | **Building Loss** | **Contents Loss** | **Use Loss: Homeowner (months)** | **Use Loss: Landlord (months)** | **Use Loss: Tenant (months)** |
| –2.0 | 0 | 0 | 0 | 0 | 0 |
| –1.5 | 0.0125 | 0.0120 | 0 | 0 | 0 |
| –1.0 | 0.0250 | 0.0240 | 0 | 0 | 0 |
| –0.5 | 0.0795 | 0.0525 | 0 | 0 | 0 |
| 0.0 | 0.1340 | 0.0810 | 9 | 10 | 1 |
| 0.5 | 0.1835 | 0.1070 | 9 | 10 | 1 |
| 1.0 | 0.2330 | 0.1330 | 9 | 10 | 1 |
| 1.5 | 0.2770 | 0.1560 | 9 | 10 | 1 |
| 2.0 | 0.3210 | 0.1790 | 9 | 10 | 1 |
| 2.5 | 0.3610 | 0.1995 | 9 | 10 | 1 |
| 3.0 | 0.4010 | 0.2200 | 9 | 10 | 1 |
| 3.5 | 0.4360 | 0.2385 | 9 | 10 | 1 |
| 4.0 | 0.4710 | 0.2570 | 12 | 13 | 1 |
| 4.5 | 0.5015 | 0.2725 | 12 | 13 | 1 |
| 5.0 | 0.5320 | 0.2880 | 12 | 13 | 1 |
| 5.5 | 0.5590 | 0.3015 | 12 | 13 | 1 |
| 6.0 | 0.5860 | 0.3150 | 12 | 13 | 1 |
| 6.5 | 0.6090 | 0.3265 | 12 | 13 | 1 |
| 7.0 | 0.6320 | 0.3380 | 12 | 13 | 1 |
| 7.5 | 0.6520 | 0.3475 | 12 | 13 | 1 |
| 8.0 | 0.6720 | 0.3570 | 15 | 16 | 1 |
| 8.5 | 0.6885 | 0.3645 | 24 | 25 | 1 |
| 9.0 | 0.7050 | 0.3720 | 24 | 25 | 1 |
| 9.5 | 0.7185 | 0.3780 | 24 | 25 | 1 |
| 10.0 | 0.7320 | 0.3840 | 24 | 25 | 1 |
| 10.5 | 0.7430 | 0.3880 | 24 | 25 | 1 |
| 11.0 | 0.7540 | 0.3920 | 24 | 25 | 1 |
| 11.5 | 0.7630 | 0.3945 | 24 | 25 | 1 |
| 12.0 | 0.7720 | 0.3970 | 24 | 25 | 1 |
| 12.5 | 0.7785 | 0.3985 | 24 | 25 | 1 |
| 13.0 | 0.7850 | 0.4000 | 24 | 25 | 1 |
| 13.5 | 0.7900 | 0.4000 | 24 | 25 | 1 |
| 14.0 | 0.7950 | 0.4000 | 24 | 25 | 1 |
| 14.5 | 0.7985 | 0.4000 | 24 | 25 | 1 |
| 15.0 | 0.8020 | 0.4000 | 24 | 25 | 1 |
| 15.5 | 0.8045 | 0.4000 | 24 | 25 | 1 |
| 16.0 | 0.8070 | 0.4000 | 24 | 25 | 1 |

**Supplementary Table 3.**  As in Supplementary Table 2, but for Two-or-More-Story Home without Basement (DDF2).

| **From USACE (2000)** | | | **From FEMA (2013)** | | |
| --- | --- | --- | --- | --- | --- |
| **Flood Depth in Structure**  **(**$\boldsymbol{dh}$**, in feet)** | **Building Loss** | **Contents Loss** | **Use Loss: Homeowner**  **(months)** | **Use Loss: Landlord**  **(months)** | **Use Loss: Tenant (months)** |
| –2.0 | 0 | 0 | 0 | 0 | 0 |
| –1.5 | 0.0150 | 0.0050 | 0 | 0 | 0 |
| –1.0 | 0.0300 | 0.0100 | 0 | 0 | 0 |
| –0.5 | 0.0615 | 0.0300 | 0 | 0 | 0 |
| 0.0 | 0.0930 | 0.0500 | 9 | 10 | 1 |
| 0.5 | 0.1225 | 0.0685 | 9 | 10 | 1 |
| 1.0 | 0.1520 | 0.0870 | 9 | 10 | 1 |
| 1.5 | 0.1805 | 0.1045 | 9 | 10 | 1 |
| 2.0 | 0.2090 | 0.1220 | 9 | 10 | 1 |
| 2.5 | 0.2360 | 0.1385 | 9 | 10 | 1 |
| 3.0 | 0.2630 | 0.1550 | 9 | 10 | 1 |
| 3.5 | 0.2885 | 0.1700 | 9 | 10 | 1 |
| 4.0 | 0.3140 | 0.1850 | 12 | 13 | 1 |
| 4.5 | 0.3380 | 0.1990 | 12 | 13 | 1 |
| 5.0 | 0.3620 | 0.2130 | 12 | 13 | 1 |
| 5.5 | 0.3845 | 0.2260 | 12 | 13 | 1 |
| 6.0 | 0.4070 | 0.2390 | 12 | 13 | 1 |
| 6.5 | 0.4280 | 0.2510 | 12 | 13 | 1 |
| 7.0 | 0.4490 | 0.2630 | 12 | 13 | 1 |
| 7.5 | 0.4685 | 0.2735 | 12 | 13 | 1 |
| 8.0 | 0.4880 | 0.2840 | 15 | 16 | 1 |
| 8.5 | 0.5060 | 0.2935 | 24 | 25 | 1 |
| 9.0 | 0.5240 | 0.3030 | 24 | 25 | 1 |
| 9.5 | 0.5405 | 0.3115 | 24 | 25 | 1 |
| 10.0 | 0.5570 | 0.3200 | 24 | 25 | 1 |
| 10.5 | 0.5720 | 0.3270 | 24 | 25 | 1 |
| 11.0 | 0.5870 | 0.3340 | 24 | 25 | 1 |
| 11.5 | 0.6005 | 0.3405 | 24 | 25 | 1 |
| 12.0 | 0.6140 | 0.3470 | 24 | 25 | 1 |
| 12.5 | 0.6260 | 0.3515 | 24 | 25 | 1 |
| 13.0 | 0.6380 | 0.3560 | 24 | 25 | 1 |
| 13.5 | 0.6485 | 0.3600 | 24 | 25 | 1 |
| 14.0 | 0.6590 | 0.3640 | 24 | 25 | 1 |
| 14.5 | 0.6680 | 0.3665 | 24 | 25 | 1 |
| 15.0 | 0.6770 | 0.3690 | 24 | 25 | 1 |
| 15.5 | 0.6845 | 0.3705 | 24 | 25 | 1 |
| 16.0 | 0.6920 | 0.3720 | 24 | 25 | 1 |

**Supplementary Table 4.** As in Supplementary Table 2, but for One-Story Home with Basement (DDF3).

| **From USACE (2000)** | | | **From FEMA (2013)** | | |
| --- | --- | --- | --- | --- | --- |
| **Flood Depth in Structure**  **(**$\boldsymbol{dh}$**, in feet)** | **Building Loss** | **Contents Loss** | **Use Loss: Homeowner**  **(months)** | **Use Loss: Landlord**  **(months)** | **Use Loss: Tenant (months)** |
| –8.0 | 0 | 0 | 9 | 10 | 1 |
| –7.5 | 0.0035 | 0.0040 | 9 | 10 | 1 |
| –7.0 | 0.0070 | 0.0080 | 9 | 10 | 1 |
| –6.5 | 0.0075 | 0.0145 | 9 | 10 | 1 |
| –6.0 | 0.0080 | 0.0210 | 9 | 10 | 1 |
| –5.5 | 0.0160 | 0.0290 | 9 | 10 | 1 |
| –5.0 | 0.0240 | 0.0370 | 9 | 10 | 1 |
| –4.5 | 0.0380 | 0.0470 | 9 | 10 | 1 |
| –4.0 | 0.0520 | 0.0570 | 12 | 13 | 1 |
| –3.5 | 0.0710 | 0.0685 | 12 | 13 | 1 |
| –3.0 | 0.0900 | 0.0800 | 12 | 13 | 1 |
| –2.5 | 0.1140 | 0.0925 | 12 | 13 | 1 |
| –2.0 | 0.1380 | 0.1050 | 12 | 13 | 1 |
| –1.5 | 0.1660 | 0.1185 | 12 | 13 | 1 |
| –1.0 | 0.1940 | 0.1320 | 12 | 13 | 1 |
| –0.5 | 0.2245 | 0.1460 | 12 | 13 | 1 |
| 0.0 | 0.2550 | 0.1600 | 15 | 16 | 1 |
| 0.5 | 0.2875 | 0.1745 | 15 | 16 | 1 |
| 1.0 | 0.3200 | 0.1890 | 15 | 16 | 1 |
| 1.5 | 0.3535 | 0.2035 | 15 | 16 | 1 |
| 2.0 | 0.3870 | 0.2180 | 15 | 16 | 1 |
| 2.5 | 0.4210 | 0.2325 | 15 | 16 | 1 |
| 3.0 | 0.4550 | 0.2470 | 15 | 16 | 1 |
| 3.5 | 0.4885 | 0.2605 | 15 | 16 | 1 |
| 4.0 | 0.5220 | 0.2740 | 15 | 16 | 1 |
| 4.5 | 0.5540 | 0.2870 | 15 | 16 | 1 |
| 5.0 | 0.5860 | 0.3000 | 15 | 16 | 1 |
| 5.5 | 0.6155 | 0.3120 | 15 | 16 | 1 |
| 6.0 | 0.6450 | 0.3240 | 18 | 19 | 1 |
| 6.5 | 0.6715 | 0.3345 | 24 | 25 | 1 |
| 7.0 | 0.6980 | 0.3450 | 24 | 25 | 1 |
| 7.5 | 0.7200 | 0.3540 | 24 | 25 | 1 |
| 8.0 | 0.7420 | 0.3630 | 24 | 25 | 1 |
| 8.5 | 0.7595 | 0.3700 | 24 | 25 | 1 |
| 9.0 | 0.7770 | 0.3770 | 24 | 25 | 1 |
| 9.5 | 0.7890 | 0.3815 | 24 | 25 | 1 |
| 10.0 | 0.8010 | 0.3860 | 24 | 25 | 1 |
| 10.5 | 0.8060 | 0.3885 | 24 | 25 | 1 |
| 11.0 | 0.8110 | 0.3910 | 24 | 25 | 1 |
| 11.5 | 0.8110 | 0.3910 | 24 | 25 | 1 |
| 12.0 | 0.8110 | 0.3910 | 24 | 25 | 1 |
| 12.5 | 0.8110 | 0.3910 | 24 | 25 | 1 |
| 13.0 | 0.8110 | 0.3910 | 24 | 25 | 1 |
| 13.5 | 0.8110 | 0.3910 | 24 | 25 | 1 |
| 14.0 | 0.8110 | 0.3910 | 24 | 25 | 1 |
| 14.5 | 0.8110 | 0.3910 | 24 | 25 | 1 |
| 15.0 | 0.8110 | 0.3910 | 24 | 25 | 1 |
| 15.5 | 0.8110 | 0.3910 | 24 | 25 | 1 |
| 16.0 | 0.8110 | 0.3910 | 24 | 25 | 1 |

**Supplementary Table 5.** As in Supplementary Table 2, but for Two-or-More-Story Home with Basement (DDF4).

| **From USACE (2000)** | | | **From FEMA (2013)** | | |
| --- | --- | --- | --- | --- | --- |
| **Flood Depth in Structure**  **(**$\boldsymbol{dh}$**, in feet)** | **Building Loss** | **Contents Loss** | **Use Loss: Homeowner**  **(months)** | **Use Loss: Landlord**  **(months)** | **Use Loss: Tenant (months)** |
| –8.0 | 0 | 0 | 9 | 10 | 1 |
| –7.5 | 0.0085 | 0.0050 | 9 | 10 | 1 |
| –7.0 | 0.0170 | 0.0100 | 9 | 10 | 1 |
| –6.5 | 0.0180 | 0.0165 | 9 | 10 | 1 |
| –6.0 | 0.0190 | 0.0230 | 9 | 10 | 1 |
| –5.5 | 0.0240 | 0.0300 | 9 | 10 | 1 |
| –5.0 | 0.0290 | 0.0370 | 9 | 10 | 1 |
| –4.5 | 0.0380 | 0.0445 | 9 | 10 | 1 |
| –4.0 | 0.0470 | 0.0520 | 12 | 13 | 1 |
| –3.5 | 0.0595 | 0.0600 | 12 | 13 | 1 |
| –3.0 | 0.0720 | 0.0680 | 12 | 13 | 1 |
| –2.5 | 0.0870 | 0.0760 | 12 | 13 | 1 |
| –2.0 | 0.1020 | 0.0840 | 12 | 13 | 1 |
| –1.5 | 0.1205 | 0.0925 | 12 | 13 | 1 |
| –1.0 | 0.1390 | 0.1010 | 12 | 13 | 1 |
| –0.5 | 0.1590 | 0.1100 | 12 | 13 | 1 |
| 0.0 | 0.1790 | 0.1190 | 15 | 16 | 1 |
| 0.5 | 0.2010 | 0.1285 | 15 | 16 | 1 |
| 1.0 | 0.2230 | 0.1380 | 15 | 16 | 1 |
| 1.5 | 0.2465 | 0.1475 | 15 | 16 | 1 |
| 2.0 | 0.2700 | 0.1570 | 15 | 16 | 1 |
| 2.5 | 0.2945 | 0.1670 | 15 | 16 | 1 |
| 3.0 | 0.3190 | 0.1770 | 15 | 16 | 1 |
| 3.5 | 0.3440 | 0.1875 | 15 | 16 | 1 |
| 4.0 | 0.3690 | 0.1980 | 15 | 16 | 1 |
| 4.5 | 0.3940 | 0.2090 | 15 | 16 | 1 |
| 5.0 | 0.4190 | 0.2200 | 15 | 16 | 1 |
| 5.5 | 0.4440 | 0.2315 | 15 | 16 | 1 |
| 6.0 | 0.4690 | 0.2430 | 18 | 19 | 1 |
| 6.5 | 0.4935 | 0.2550 | 24 | 25 | 1 |
| 7.0 | 0.5180 | 0.2670 | 24 | 25 | 1 |
| 7.5 | 0.5410 | 0.2790 | 24 | 25 | 1 |
| 8.0 | 0.5640 | 0.2910 | 24 | 25 | 1 |
| 8.5 | 0.586 | 0.3040 | 24 | 25 | 1 |
| 9.0 | 0.6080 | 0.3170 | 24 | 25 | 1 |
| 9.5 | 0.6280 | 0.3305 | 24 | 25 | 1 |
| 10.0 | 0.6480 | 0.3440 | 24 | 25 | 1 |
| 10.5 | 0.6660 | 0.3580 | 24 | 25 | 1 |
| 11.0 | 0.6840 | 0.3720 | 24 | 25 | 1 |
| 11.5 | 0.6990 | 0.3860 | 24 | 25 | 1 |
| 12.0 | 0.7140 | 0.4000 | 24 | 25 | 1 |
| 12.5 | 0.7255 | 0.4150 | 24 | 25 | 1 |
| 13.0 | 0.7370 | 0.4300 | 24 | 25 | 1 |
| 13.5 | 0.7455 | 0.4455 | 24 | 25 | 1 |
| 14.0 | 0.7540 | 0.4610 | 24 | 25 | 1 |
| 14.5 | 0.7590 | 0.4770 | 24 | 25 | 1 |
| 15.0 | 0.7640 | 0.4930 | 24 | 25 | 1 |
| 15.5 | 0.7640 | 0.5095 | 24 | 25 | 1 |
| 16.0 | 0.7640 | 0.5260 | 24 | 25 | 1 |

**Supplementary Table 6.** Output Parameters for the 29 Homes in the Metairie, Louisiana, Study

Area.

| **Home Number** | $\boldsymbol{AAL}_{\boldsymbol{B}\boldsymbol{/}\boldsymbol{V}_{\boldsymbol{R}}}$ | $\boldsymbol{AAL}_{\boldsymbol{C}\boldsymbol{/}\boldsymbol{V}_{\boldsymbol{R}}}$ | $\boldsymbol{AAL}_{\boldsymbol{B}\boldsymbol{\$}}$ | $\boldsymbol{AAL}_{\boldsymbol{C}\boldsymbol{\$}}$ | $\boldsymbol{AAL}_{\boldsymbol{UH}\boldsymbol{\$}}$ | $\boldsymbol{AAL}_{\boldsymbol{UL}\boldsymbol{\$}}$ | $\boldsymbol{AAL}_{\boldsymbol{UT}\boldsymbol{\$}}$ | $\boldsymbol{AAL}_{\boldsymbol{H}\boldsymbol{\$}}$ | $\boldsymbol{AAL}_{\boldsymbol{L}\boldsymbol{\$}}$ | $\boldsymbol{AAL}_{\boldsymbol{T}\boldsymbol{\$}}$ |
| --- | --- | --- | --- | --- | --- | --- | --- | --- | --- | --- |
| 1 | 0.01148257 | 0.007882995 | 3536 | 2428 | 736 | 817 | 97 | 6700 | 4354 | 2525 |
| 2 | 0.012291487 | 0.008280812 | 3304 | 2226 | 859 | 955 | 130 | 6389 | 4259 | 2356 |
| 3 | 0.012013395 | 0.008142757 | 2688 | 1822 | 656 | 729 | 119 | 5166 | 3417 | 1941 |
| 4 | 0.01148257 | 0.007882995 | 3723 | 2556 | 774 | 860 | 97 | 7052 | 4583 | 2653 |
| 5 | 0.011996987 | 0.005688878 | 2779 | 1318 | 848 | 942 | 149 | 4945 | 3721 | 1467 |
| 6 | 0.00582355 | 0.003858238 | 1555 | 1030 | 466 | 518 | 71 | 3052 | 2073 | 1101 |
| 7 | 0.005712388 | 0.003757612 | 1057 | 695 | 333 | 370 | 73 | 2086 | 1427 | 768.5 |
| 8 | 0.005520101 | 0.003643479 | 1065 | 703 | 328 | 365 | 69 | 2097 | 1430 | 772.2 |
| 9 | 0.004047217 | 0.002610190 | 596 | 298 | 278 | 309 | 58 | 894 | 905 | 367 |
| 10 | 0.005196178 | 0.003387004 | 1323 | 509 | 278 | 309 | 58 | 2627 | 1814 | 932.9 |
| 11 | 0.005363381 | 0.002589281 | 1471 | 710 | 536 | 595 | 79 | 2717 | 2066 | 789.5 |
| 12 | 0.006973243 | 0.004663137 | 1304 | 872 | 363 | 403 | 79 | 2539 | 1707 | 950.9 |
| 13 | 0.018980867 | 0.013030256 | 4086 | 2805 | 853 | 948 | 161 | 7744 | 5034 | 2966 |
| 14 | 0.014873246 | 0.010146573 | 3141 | 2143 | 711 | 790 | 137 | 5995 | 3931 | 2280 |
| 15 | 0.011743161 | 0.008009775 | 2926 | 1996 | 663 | 737 | 108 | 5584 | 3662 | 2104 |
| 16 | 0.007181083 | 0.004869160 | 1827 | 1239 | 443 | 492 | 71 | 3509 | 2319 | 1310 |
| 17 | 0.008079082 | 0.005445602 | 2074 | 1398 | 536 | 595 | 85 | 4008 | 2670 | 1483 |
| 18 | 0.004978243 | 0.002439908 | 1829 | 896 | 751 | 835 | 83 | 3476 | 2664 | 979.3 |
| 19 | 0.004315560 | 0.002795365 | 663 | 330 | 302 | 335 | 64 | 1295 | 998 | 394 |
| 20 | 0.00594076 | 0.003904417 | 1152 | 757 | 365 | 406 | 77 | 2274 | 1558 | 833.5 |
| 21 | 0.02432078 | 0.016682273 | 6902 | 4735 | 1460 | 1623 | 209 | 13097 | 8525 | 4943 |
| 22 | 0.011743161 | 0.008009775 | 2132 | 1454 | 483 | 537 | 108 | 4068 | 2668 | 1562 |
| 23 | 0.007667654 | 0.005128669 | 2211 | 1479 | 614 | 682 | 86 | 4303 | 2893 | 1565 |
| 24 | 0.012013395 | 0.008142757 | 4096 | 2776 | 999 | 1110 | 119 | 7872 | 5206 | 2895 |
| 25 | 0.007146793 | 0.004761343 | 1254 | 835 | 360 | 400 | 83 | 2449 | 1654 | 918.7 |
| 26 | 0.006570546 | 0.004340031 | 1503 | 993 | 461 | 512 | 82 | 2957 | 2015 | 1075 |
| 27 | 0.01148257 | 0.007882995 | 2613 | 1794 | 543 | 604 | 97 | 4950 | 3216 | 1891 |
| 28 | 0.009599022 | 0.004552596 | 2366 | 1122 | 722 | 802 | 119 | 4210 | 3168 | 1241 |
| 29 | 0.009627856 | 0.006523038 | 1583 | 1072 | 388 | 431 | 96 | 3043 | 2014 | 1168 |
